# PH-Sensitive, Polymer Functionalized, Nonporous Silica Nanoparticles for Quercetin Controlled Release

**DOI:** 10.3390/polym11122026

**Published:** 2019-12-06

**Authors:** Lin Xu, Hong-Liang Li, Li-Ping Wang

**Affiliations:** 1 College of Materials Science and Engineering, Qingdao University, Qingdao 266071, China; xulinbang@163.com; 2 College of Materials Science and Engineering, Liaocheng University, Liaocheng 252059, China

**Keywords:** silica nanoparticles, PDEAEMA, metal-free ATRP, quercetin, controlled drug release

## Abstract

Some pH-sensitive, poly(2-(diethylamino)ethyl methacrylate) (PDEAEMA) grafted silica nanoparticles (SNPs) (SNPs-*g*-PDEAEMA) were designed and synthesized via surface initiated, metal-free, photoinduced atom transfer radical polymerization (ATRP). The structures of the polymers formed in solution were determined by ^1^H NMR. The modified nanoparticles were characterized by FT-IR spectroscopy, XPS, GPC, TEM and TGA. The analytical results show that α-bromoisobutyryl bromide (BIBB) (ATRP initiator) had been successfully anchored onto SNPs’ surfaces, and was followed by surface-initiated, metal-free ATRP of 2-(diethylamino)ethyl methacrylate (DEAEMA). The resultant SNPs-*g*-PDEAEMA were uniform spherical nanoparticles with the particles size of about 22–25 nm, and the graft density of PDEAEMA on SNPs’ surfaces obtained by TGA was 19.98 μmol/m^2^. Owing to the covalent grafting of pH-sensitive PDEAEMA, SNPs-*g*-PDEAEMA can dispersed well in acidic aqueous solution, but poorly in neutral and alkaline aqueous solutions, which is conducive to being employed as drug carriers to construct a pH-sensitive controlled drug delivery system. In vitro cytotoxicity evaluation results showed that the cytotoxicity of SNPs-*g*-PDEAEMA to the L929 cells had completely disappeared on the 3rd day. The loading of quercetin on SNPs-*g*-PDEAEMA was performed using adsorption process from ethanol solutions, and the dialysis release rate increased sharply when the pH value of phosphate-buffered saline (PBS) decreased from 7.4 to 5.5. All these results indicated that the pH-responsive microcapsules could serve as potential anti-cancer drug carriers.

## 1. Introduction

In recent years, various inorganic-organic hybrid nanomaterials have come out as ideal drug carriers for controlled release systems [1,2,3,4,5]. In this way, inorganic nanomaterials provide a robust framework, while integrated organic components offer functionality. These existing inorganic nanomaterials, such as silica nanoparticles [4,5], TiO_2_ nanotubes [6] and ZnO nanoparticles [7], not only conquer the problem of drug delivery, but also improve the therapeutic efficiency of drugs. Silica nanoparticles (SNPs) are considered a carrier of drugs owing to their excellent properties such as large specific surface area, high chemical and mechanical stability, prominent biocompatibility, well-defined surface chemistry and low cytotoxicity [8,9]. Moreover, organic components serve as the ‘‘net’’ of drugs, which can release the encapsulated molecules just as in a specific environment stimulated by the outside world. Meanwhile, pH-sensitive systems have received particular attention in a variety of application stimuli, as it has been shown that the pH values of tumor tissues (6.5–7.0) are lower those that of bloodstream and normal tissues (7.4), and the pHs of endosomes and lysosomas are much lower (5.0–6.5). Therefore, pH-responsive controlled release containers are very necessary in practical applications [10,11,12,13,14].

Surface-initiated atom transfer radical polymerization (SI-ATRP) [15,16,17,18,19,20] is an important synthetic means for preparing various inorganic-polymer hybrid nanomaterials. However, traditional ATRP requires a transition-metal catalyst (such as Cu^+^ and Fe^2+^) to retain activity throughout the polymerization process, leading to unavoidable metal residues in the final product and impeded applications in some biomedical or electronic fields [21,22,23]. Photoinduced metal-free ATRP was first reported to solve this drawback in 2014 [24]. In this process, 10-phenylphenothiazine (PTH), an organic-based photoredox catalyst replaced the transition-metal catalyst and is mediated by light; thus, the toxicity of metal catalyst is avoided. Since then, photoinduced, metal-free ATRP has been successfully used as the surface modification of different inorganic materials, such as nanodiamond (ND) [25], SBA-15 [26] and silica [27].

Quercetin (Qu) is the most abundant bioflavonoid with interesting biological properties, such as antiviral, antiallergenic, anti-inflammatory, antibacterial and anticancer activities [8]. The clinical trials have confirmed Qu’s effective anticancerous properties and applied it to the treatment of tumor tissues such as those of the colon, liver, brain and prostate to inhibit the spread of malignant cells [28]. Although quercetin is good for the health, its poor bioavailability limits its therapeutic method. The low bioavailability of Qu is primary due to its restricted absorption capacity (low stability, poor water solubility, short half-life) and its rapid elimination [9]. 

**Scheme 1:**
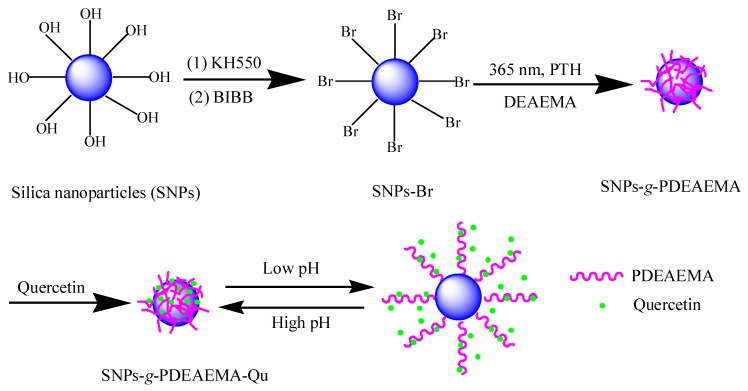
Synthesis of SNPs-*g*-PDEAEMA, scheme of drug loading and pH-dependent release from SNPs-*g*-PDEAEMA.

The primary objective of this study was to design a novel pH-sensitive, inorganic–organic hybrid nanomaterials via photoinduced, metal-free SI-ATRP for Qu controlled release. PDEAEMA was selected to be grafted from SNPs’ surfaces for its pH-sensitive and amphiphilic structure; its solubility varies greatly between water soluble and water insoluble corresponding to the protonation and deprotonation of the amine groups on the side depending on the pH values [12]. Under normal physiological conditions (pH = 7.4), the self-assembled Qu-loaded microcapsules have tight and compact structures and the drugs are entrapped in the cores. Meanwhile, in weak acid or neutral conditions, the drug-loaded microcapsules become loose and swollen due to the protonation of the amine groups on PDEAEMA, leading to drug release from the drug-loaded micelles. The detailed reaction and controlled release processes are listed in Scheme 1.

## 2. Experimental Procedures

### 2.1. Synthesis of SNPs-g-PDEAEMA

SNPs-Br was prepared according to the procedure described in previous study [29]. XPS calculated (atomic %): Si, 22.05; C, 25.16; N, 5.31; O, 44.22; Br, 3.27; Br, 1.67 mmol·g^−1^ (calculated according to the Br atomic %) (Table 1). SNPs-*g*-PDEAEMA was synthesized via photoinduced metal-free SI-ATRP. Briefly, DEAEMA (2 mL, 10 mmol), SNPs-Br (0.15 g, 0.250 mmol Br groups), Ethyl 2-bromoisobutyrate (5 μL, 0.031 mmol), PTH (0.009 g, 0.033 mmol), 1.6 mL ethanol and 0.4 mL deionized water were mixed in a 10 mL quartz tube with a magnetic stirrer. The mixture was stirred for 20 min at room temperature in the dark. After dispersed uniformly, the reaction mixture was irradiated under a 365 nm ultraviolet lamp at room temperature in dark for 4 h. The reaction mixture was centrifuged and separated into two parts: a clear liquid at the upper layer (mainly contained the PDEAEMA homopolymer) and precipitation at the bottom (mainly contained crude SNPs-*g*-PDEAEMA). PDEAEMA homopolymer was obtained by precipitation of the clear liquid in water. In addition, the crude SNPs-*g*-PDEAEMA was precipitated in water and dried at room temperature under vacuum. Subsequently, the crude SNPs-*g*-PDEAEMA was purified with ethanol via Soxhlet extraction for 12 h to get rid of the ungrafted PDEAEMA completely, and dried in vacuum at room temperature. The detailed reaction process is shown in Scheme 1.

### 2.2. Quercetin Loading

Quercetin loading were performed according to previously described literature [30,31,32,11]. Typically, quercetin (0.02 g) and SNPs-*g*-PDEAEMA (0.02 g) in weight ratio 1:1 were stirred in ethanol (1 mL) until the solvent completely evaporated. Then, the powdered products were washed three times with water (5 mL) to remove the physically absorbed quercetin and dried overnight at 30 °C. The quercetin loaded SNPs-*g*-PDEAEMA formulation was designated as SNPs-*g*-PDEAEMA-Qu. The amount of loaded quercetin for SNPs-*g*-PDEAEMA was monitored by a fluorescence spectrophotometer at 367 nm. The drug concentration was calculated according to the standard curve of pure quercetin dissolved in ethanol. The drug loading capacity (33.82%) and entrapment efficiency (67.64%) were calculated using the following formula.

(1) $$\mathrm{Drug}\;\mathrm{loading}\;\left(\%\right)=\frac{\mathrm{weight}\;\mathrm{of}\;\mathrm{drug}\;\mathrm{in}\;\mathrm{SNPs}-g-\mathrm{PDEAEMA}\;}{\mathrm{weight}\;\mathrm{of}\;\mathrm{SNPs}-g-\mathrm{PDEAEMA}-\mathrm{Qu}}\;\times \;100\%$$

(2) $$\mathrm{Entrapment}\;\mathrm{efficiency}\;\left(\%\right)=\frac{\mathrm{weight}\;\mathrm{of}\;\mathrm{drug}\;\mathrm{in}\;\mathrm{SNPs}-g-\mathrm{PDEAEMA}\;}{\mathrm{initial}\;\mathrm{weight}\;\mathrm{of}\;\mathrm{drug}}\;\times \;100\%$$

### 2.3. In Vitro Cytotoxicity Evaluation

According to ISO 10993-5 Standard and previously literature [33,34,35], the in vitro cytotoxicity of SNPs-*g*-PDEAEMA was evaluated on an extracted solution of SNPs-*g*-PDEAEMA via MTT assay. In Briefly, the sterilized SNPs-*g*-PDEAEMA was extracted using DMEM at an extraction ratio of 0.5 mg·mL^−1^ for 24 h at 37 °C. The medium (100 μL) containing 10^4^ L929 cells and extract solution (100 μL) was plated into each well of the 96-well plate and then incubated in 5% CO_2_ atmosphere at 37 °C. MTT (20 μL, 5 mg·mL^−1^ in PBS) was added for 4 h and allowing the formation of formazan crystals on the 1st, 2nd and 3rd days. After removing the supernatant, 150 μL DMSO was added to each well, and the absorbance was monitored by a microplate reader at 490 nm. The data were expressed as percentages relative to the data obtained with blank control. Each group was tested with six samples.

### 2.4. Cell Uptake Assay

L929 cells were inoculated on the focusing plate with a cellular concentration of 1 × 10^5^, and incubated for 24 h. In total, 10 ug/ml of cy5.5-labeled SNPs-*g*-PDEAEMA-Qu (SNPs-*g*-PDEAEMA-Qu-cy5.5) was added, and the resulting solution was incubated for 0.5, 2 and 4 h. Cell nucleus were stained with blue fluorescence by Hoechst 33258. Cell uptake images were recorded using an ix-71 inverted fluorescence microscope (Olympus, Japan).

### 2.5. In Vitro Release of Quercetin from SNPs-g-PDEAEMA

In vitro quercetin drug release research was carried out in buffer solution with pH values of 5.5 and 7.4 at 37 °C. In a typical experiment, 2.0 mg of SNPs-*g*-PDEAEMA-Qu was suspended in 3.0 mL of phosphate-buffered saline (PBS) (pH 5.5 or 7.4). The resulting solution was moved to a dialysis bag (molecular weight cutoff 7500–8000), placed in conical flasks containing 97 mL of PBS and vibrated at thermostatic water bath oscillator. At appropriate time interval, 3 mL solution samples (*V*_e_) were removed from the release medium and an equal volume of fresh buffer was added to maintain the total volume. The release of quercetin in different PBS was monitored via UV–Vis spectroscopy at the wavelength of 367 nm. The cumulative percentage of drug release (*E*_r_) was calculated according to the calibration curve (r > 0.999) and the following Equation (3).

(3) $$Er\;\left(\%\right)=\frac{V_{e}\sum_{1}^{n-1}C_{i}+V_{0}C_{n}}{m_{Q_{u}}}\;\;\times \;100\%$$

where *m_Qu_* is the amount of quercetin in the SNPs-*g*-PDEAEMA, Cn is the concentration of quercetin in the *n_th_* sample, *V*_0_ represents the volume of the release medium (*V*_0_ = 100 mL). The in vitro release of quercetin was performed in triplicate at different pH conditions to gain the final in vitro release curves. 

## 3. Results and Discussion

### 3.1. Material Characterization

#### 3.1.1. Structure Characterization

**Figure 1:**
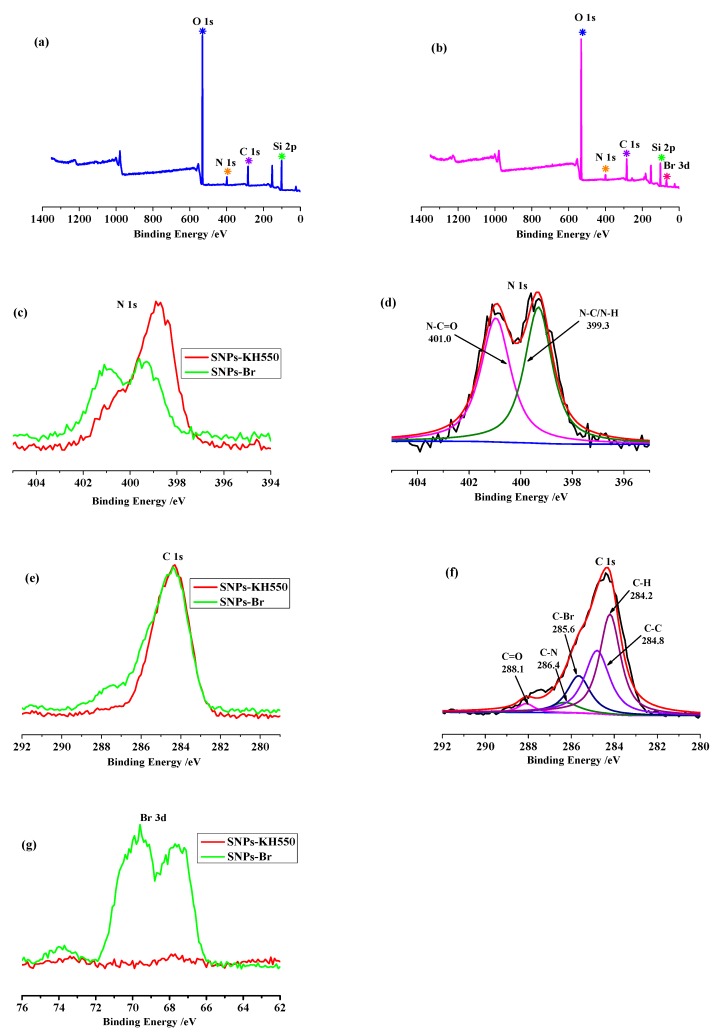
XPS wide-scan spectra of silica nanoparticles (SNPs)-KH550 (**a**) and SNPs-Br (**b**). XPS high resolution spectra of (**c**) N1s of SNPs-KH550 and SNPs-Br, (**d**) N1s core-level spectra of SNPs-Br, (**e**) C1s of SNPs-KH550 and SNPs-Br, (**f**) C1s core-level spectra of SNPs-Br and (**g**) Br3d of SNPs-KH550 and SNPs-Br.

The immobilization ATRP initiator onto SNPs’ surfaces is necessary for prepare the polymer brushes grafted from SNPs’ surfaces by metal-free SI-ATRP. The chemical compositions of SNPs-KH550 and SNPs-Br were measured by XPS analysis. Figure 1a shows the wide-scan XPS spectra of SNPs-KH550. The signals of N1s (398.7 eV) and C_1s_ (281.5 eV) were attributed to the N and C of KH550 on SNPs surface. The still-observed peaks for Si_2p_ (101.2 eV) were ascribed to the exposed Si element of SNPs. As for SNPs-Br (Figure 1b), except for the main signals of O 1s, N 1s, C 1s and Si 2p, were still observed at 533.6, 400.2, 283.3 and 102.8 eV (Figure 1b–f); a new signal of Br element appeared at 69.3 eV (Figure 1b,g). In addition, the N 1s core-level spectra of SNPs-Br showed two peak components at 399.3 eV and 401.0 eV (Figure 1d). The peak at 399.3 eV was ascribed to N–C (sp^3^) and the peak at the 401.0 eV corresponds to N–C=O bonds. The C 1s core-level spectra of SNPs-Br consisted with five types bond, including C–C, C–H, C–N, C=O and C–Br, accompanied by the appearance of peaks at 284.8, 284.2, 286.4, 288.1 and 285.6 eV, respectively (Figure 1f). These results indicated successful immobilizing of BIBB initiators onto SNPs’ surfaces. The existence of Br provided the prerequisite conditions for subsequent metal-free SI-ATRP.

**Figure 2:**
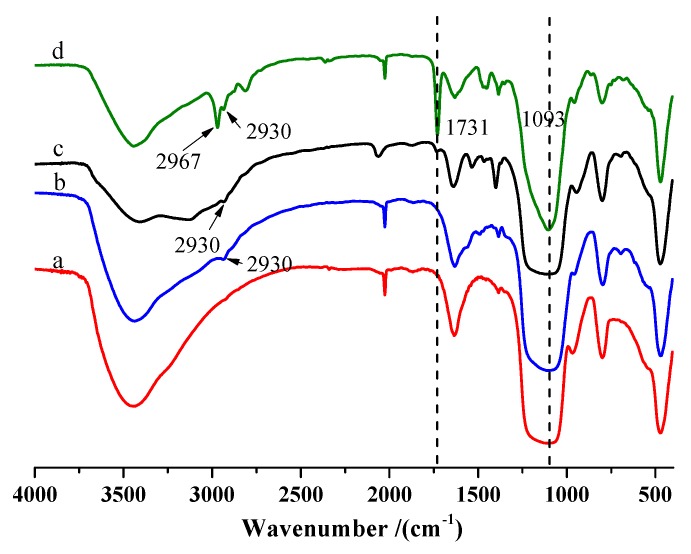
FT-IR spectra of (**a**) SNPs, (**b**) SNPs-KH550, (**c**) SNPs-Br and (**d**) SNPs-*g*-PDEAEMA.

The FT-IR spectra of SNPs, SNPs-KH550, SNPs-Br and SNPs-*g*-PDEAEMA are shown in Figure 2. The adsorption peaks at 1093 cm^−1^ were ascribed to the Si–O–Si stretching vibration of SNPs. Compared with crude SNPs, the FT-IR spectra of SNPs-KH550 (Figure 2b) shows a new characteristic peak at 2930 cm^−1^ which was attributed to CH_2_ stretching vibration of KH550 on the SNP surface. As for the SNPs-Br (Figure 2c), a new characteristic peak appeared at 1731 cm^−1^, which can be assigned to the stretching vibration of C=O in BiBB, indicating the successful amination of acyl bromide groups and amino groups. After metal free SI-ATRP (Figure 2d), the greatly enhancive peak at 1731 cm^−1^ was attributed to the stretching vibration of C=O, which is the result of the C=O overlap of BiBB and PDEAEMA. The FT-IR results illustrate the successful grafting of PDEAEMA from SNPs surface via metal-free SI-ATRP. In addition, the molecular weight and distribution of the cleaved PDEAEMA from SNPs surface determined by GPC further supported the presence of PDEAEMA grafted from SNPs surface (see Table S1).

**Figure 3:**
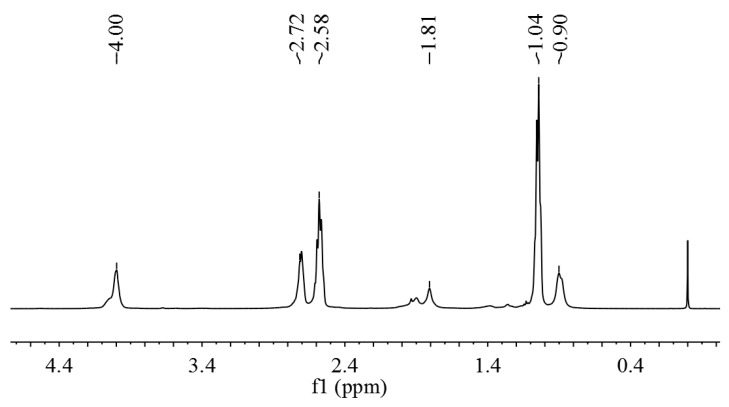
^1^H NMR spectrum of PDEAEMA formed in solution.

As a significant analytical means, the ^1^HNMR spectrum can adequately analyze the structure of PDEAEMA formed in solution (Figure 3). The signal at 0.00 ppm belongs to the NMR internal reference (TMS). The signal at 4.00 ppm was ascribed to the proton peak of –OCH_2_, and 2.72 ppm was the proton peak of CH_2_ connected with OCH_2_ in PDEAEMA. The signal at 2.58 ppm was assigned to the proton peak of NCH_2_ connected with the branch chain, and 1.04 ppm was due to the proton peak of CH_3_ connected with NCH_2_. The signals at 0.90 and 1.81 ppm were attributed to the repeating units of methyl (CH_3_) and methylene (CH_2_) in the main polymer chain. The H^1^ NMR results indicate that metal free SI-ATRP reaction was successful.

#### 3.1.2. Morphology Analysis

**Figure 4:**
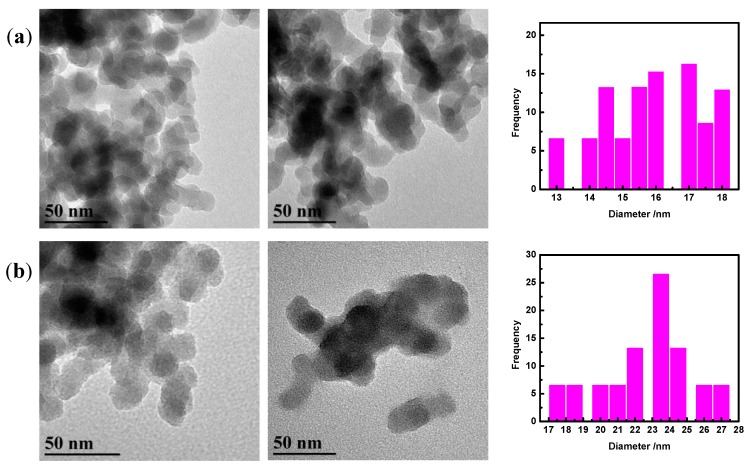
TEM images of (**a**) SNPs, (**b**) SNPs-*g*-PDEAEMA and the particle size distribution.

To get more information about the effect of PDEAEMA grafting from the SNPs surface, TEM and Nano Measurer software were used to investigating the surface topography and particle size and distribution in detail. Figure 4 presents the TEM images of SNPs and SNPs-*g*-PDEAEMA. It can be seen that the particles’ sizes exhibit a tendency of increasing with the process of grafting polymerization. The particle sizes of pure SNPs are mainly concentrated at 13–18 nm. The smaller particle size of the particles owe themselves to the higher specific surface energy, which makes them tend to aggregate and difficult to disperse (Figure 4a). This phenomenon impeded the application and development of SNPs. But after grafting PDEAEMA from SNPs surface by metal-free ATRP (Figure 4b), the particle size of SNPs-*g*-PDEAEMA increased (it was mainly distributed at 22–25 nm) and the dispersion degree was also improved. This is due to the amphiphilic PDEAEMA grafted from SNPs surfaces’ allowing them to interact with each other to promote dispersion.

#### 3.1.3. TGA and DSC Analysis

**Figure 5:**
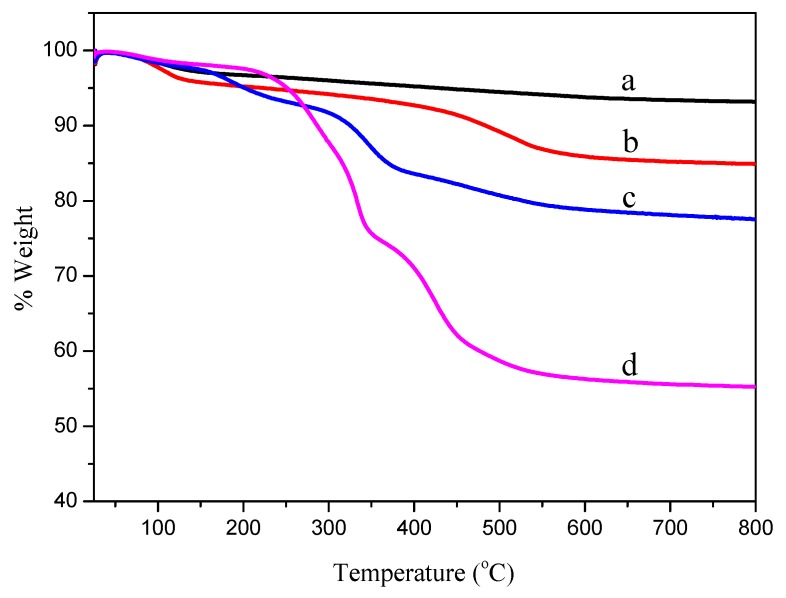
TGA curves of SNPs (**a**), SNPs-KH550 (**b**), SNPs-Br (**c**) and SNPs-*g*-PDEAEMA (**d**).

The grafting percentage of PDEAEMA from SNPs surface and the thermostability of the samples were determined by TGA. Figure 5 exhibits the TGA curves of SNPs, SNPs-KH550, SNPs-Br and SNPs-*g*-PDEAEMA with the temperature rising from 25 to 800 °C. The weight losses of SNPs, SNPs-KH550, SNPs-Br and SNPs-*g*-PDEAEMA were 6.80%, 15.20%, 22.38% and 44.86%, respectively. The graft density G_φ_ in μmol/m^2^ and molecules/nm^2^, and the percent of functionalization or level of surface coverage (f), for all functionalized materials, can be calculated via the weight loss using Equation 4 reported by Bonilla-Cruz et al [36]. The values obtained are listed in Table 2. That is, the graft density of PDEAEMA on SNPs surface is 19.98 μmol/m^2^. Figure S1 shows DSC curves of PDMAEMA and SNPs-*g*-PDMAEMA. As can be seen in Figure S1, the experimental values of *T*_g_ obtained for PDEAEMA and SNPs-*g*-PDMAEMA were 22.5 and 31.5 °C respectively. The increased *T*_g_ of PDMAEMA in SNPs-*g*-PDMAEMA, compared to pure PDMAEMA, probably caused by the confinement effect of SNPs leading to the inhibition of the chain movement of PDEAEMA.

(4) $$G\varphi_{1}\left(\frac{\mu \mathrm{mol}}{m^{2}}\right)=\frac{\frac{W_{60-800}}{100-W_{60-800}}\times 100-W_{\mathrm{SNPs}}}{M_{n}\times S_{\mathrm{spec}}\times 100}\times 10^{6}$$

where W_SNPs_ is the weight loss of SNPs (dihydroxylation) before functionalization, and W_60−800_ is the weight loss between 60 and 800 °C corresponding to the thermal decomposition of KH550, BIBB and PDEAEMA respectively. Mn is the molecular weight of the degradable part of each of the grafted KH550, BIBB and PDEAEMA, respectively. S_spec_ is the specific surface area of an SNP.

#### 3.1.4. Dispersibility and pH-Responsive Properties Analysis

**Figure 6:**
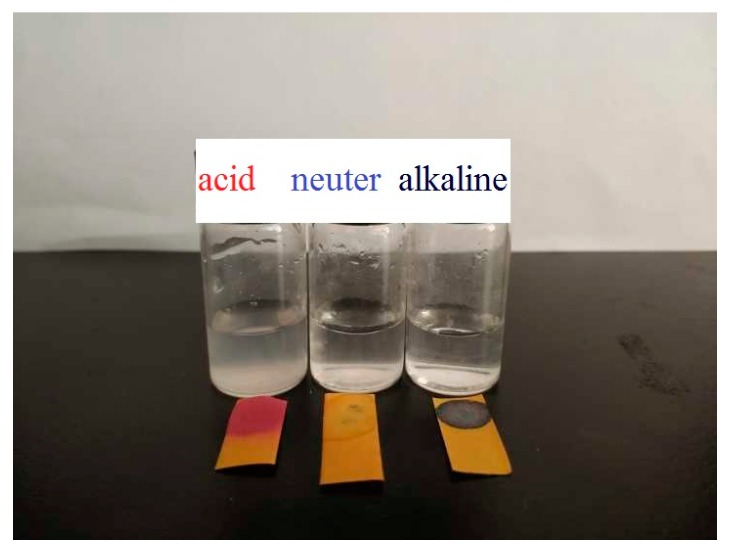
The dispersibility of SNPs-*g*-PDEAEMA in acid, neuter and alkaline aqueous solutions, respectively.

Figure 6 exhibits the images of the dispersions of SNPs-*g*-PDEAEMA in acid, neuter and alkaline aqueous solutions. It can be observed, obviously, that the hybrid materials are dispersed in acid but gathered in neuter and alkaline aqueous solutions. This is because PDEAEMA contains tertiary amine group, which is a weak base. Under acidic conditions, tertiary amine groups are protonized, and the polymer chain stretches due to mutual repulsion between electric charges. Under neutral and alkaline conditions, the protonation of groups is weakened or disappeared, the mutual repulsion between the charges on the polymer chains is weakened and the attraction between the polymers is enhanced, so the polymer chains curl and contract in water. This result further illustrated that the pH-responsive monomer DEAEMA successfully grafted from SNPs surface. 

### 3.2. In Vitro Cytotoxicity Evaluation and Cell Uptake Assay

**Figure 7:**
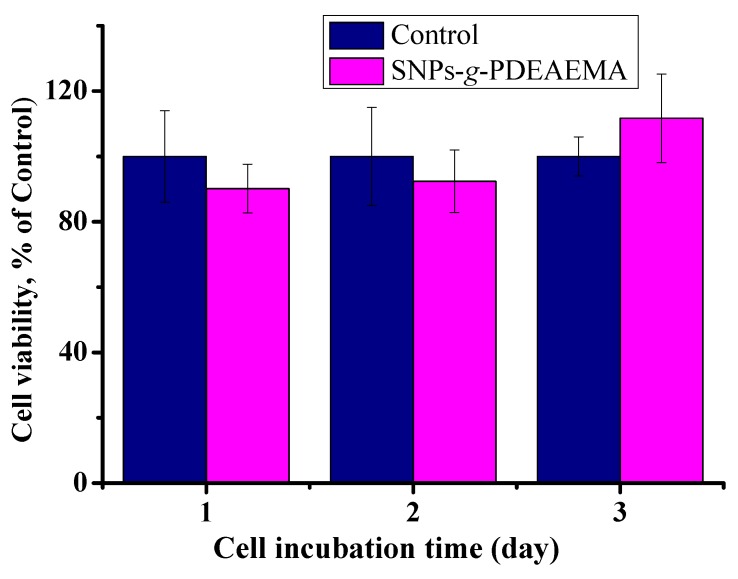
In vitro cytotoxicity of control sample and SNPs-*g*-PDEAEMA towards L929 cells.

The in vitro cytotoxicity of SNPs-*g*-PDEAEMA and a control sample were evaluated by MTT assay using L929 cells incubated with the extracted solution of SNPs-*g*-PDEAEMA for 1, 2 and 3 days. The results of quantitative evaluation of the cytotoxicity are shown in Figure 7. As shown in Figure 7, on the 1st and 2nd day, the cell viabilities were 90.18% and 92.43%, respectively; on the 3rd day, the cytotoxicity of SNPs-*g*-PDEAEMA to the L929 cells had completely disappeared, which can be ascribed to the biocompatible nature of SNPs-*g*-PDEAEMA. The value of the relative growth rate (RGR), confirmed the biocompatibility of the SNPs-*g*-PDEAEMA, sufficiently meets the requirements of the subsequent biomedical applications. 

**Figure 8:**
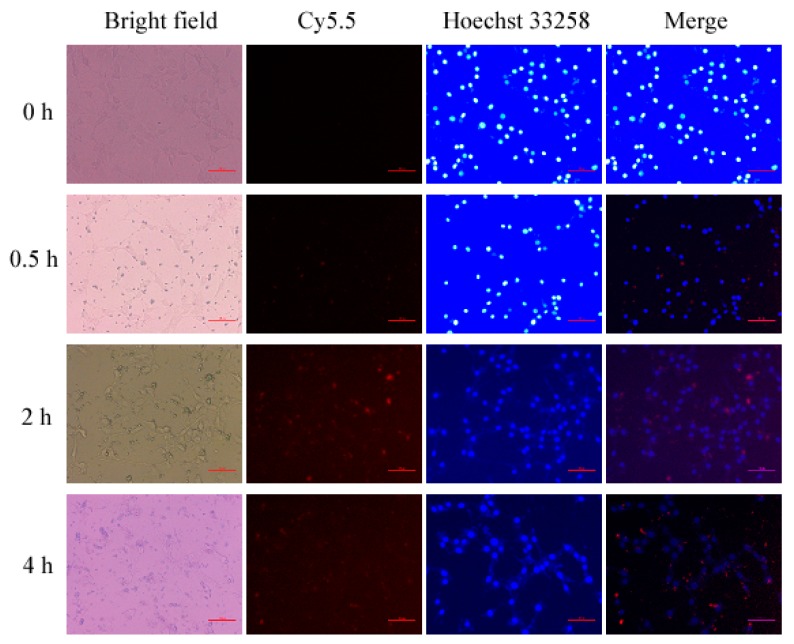
Fluorescence microscope images of L929 cells treated with SNPs-*g*-PDEAEMA-Qu-cy5.5 for 0.5, 2 and 8 h, while non-treated cells were used as the control (0 h).

To observe the cell uptake of SNPs-*g*-PDEAEMA-Qu, a fluorescence microscope (FM) was employed to track the red fluorescence of SNPs-*g*-PDEAEMA-Qu (labeled by Cy5.5) in L929 cells (Figure 8). As can be seen from Figure 8, cy5.5-labeled SNPs-*g*-PDEAEMA-Qu (SNPs-*g*-PDEAEMA-Qu-cy5.5) showed red fluorescence, cell nuclei exhibited blue fluorescence after staining by Hoechst 33258 and SNPs-*g*-PDEAEMA-Qu-cy5.5 was successfully absorbed into the cytoplasm around the cell nucleus. Moreover, the uptake is time-dependent and increases with the extension of time, indicating that SNPs-*g*-PDEAEMA as a drug carrier can be endocytosis by cells; thus, promoting drug absorption.

### 3.3. Quercetin Loading and In Vitro Release

**Figure 9:**
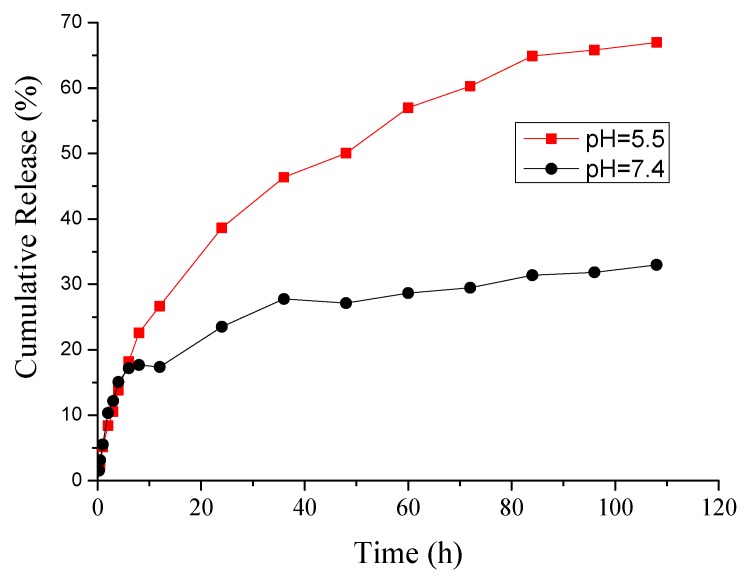
In vitro quercetin release curves of SNPs-*g*-PDEAEMA-Qu at different pH values.

In order to assess the effect of pH-sensitive properties on controlled drug release, in vitro quercetin release behavior of SNPs-*g*-PDEAEMA-Qu was measured under in a slightly acidic environment (PBS, pH 5.5) and physiological conditions (PBS, pH 7.4). Figure 9 shows the drug release curve of SNPs-*g*-PDEAEMA-Qu in buffer solution (PBS, pH 5.5 or 7.4, 37 °C). It can be observed that the release amount significantly sped up when the pH value decreased from 7.4 to 5.5. At pH 7.4, about 33% of quercetin was released in vivo within 108 h, which indicates that the drug can be well protected and stays stable with minimal quercetin release from SNPs-*g*-PDEAEMA at normal physiological conditions. At pH 5.5, about 67% of the quercetin is released at 108 h. Therefore, the release of quercetin is pH-responsive; when the pH value decreased from 7.4 to 5.5, protonated PDEAEMA chains repulsed each other electrostatically and extended, accelerating the release behavior of quercetin efficiently. An acidic environment can simulate the environment of tumor tissue or the human stomach, while an alkaline one can simulate the environment of normal tissues or the large intestine. Therefore, pH-responsive SNPs-*g*-PDEAEMA is expected to be used in clinical trials to control drug release behavior.

## 4. Conclusions

In summary, pH-responsive SNPs-*g*-PDEAEMA via metal free SI-ATRP has been successfully synthesized. The α-bromoisobutyryl bromide (BIBB) (ATRP initiator) was immobilized onto SNPs’ surfaces to initiate metal-free photoinduced SI-ATRP of DEAEMA. The morphology and chemical composition of the SNPs-*g*-PDEAEMA were verified well by TEM, FT-IR, ^1^HNMR, GPC and TGA. These results demonstrated that PDEAEMA is successfully grafted from the SNPs surfaces. For its pH-responsive properties, Qu is loaded into SNPs-*g*-PDEAEMA via an adsorption process from ethanol solutions. The in vitro release displayed pH dependence, showing a significant increase in rate as the pH value decreased from 7.4 to 5.5. Acidic conditions can simulate the environment of tumor tissues, while alkaline conditions can simulate the environment of normal tissues. Therefore, the pH-responsive SNPs-*g*-PDEAEMA is expected to be used in clinical trials to control drug release behavior.

## Figures and Tables

**Table 1:** The element compositions (atomic %) of SNPs-KH550 and SNPs-Br from XPS results.

	Elements	O	Si	N	C	Br
Sample						
SNPs-KH550		47.09	25.75	5.77	21.40	0
SNPs-Br		44.22	22.05	5.31	25.16	3.27

**Table 2:** The TGA analysis of SNPs grafted with different organics.

Sample	^a^ G_φ1_ (μmol/m^2^)	^b^ G_φ2_ (molecules/nm^2^)	^c^ f (%)
SNPs-KH550	3.05	1.84	62.79
SNPs-Br	3.59	2.17	74.06
SNPs-*g*-PDEAEMA	19.98	12.03	419.79

^a^ G_φ1_ (μmol/m^2^) was calculated using Equation (1). ^b^ G_φ2_ (molecules/nm2) = G_φ1_ × N_A_ × 10^−24^. ^c^ f (%) = (G_φ2_/2.93) × 100.

## Supplementary Materials

The supplementary materials are available online at https://www.mdpi.com/2073-4360/11/12/2026/s1.
