# Supplementary material for: PH-Sensitive, Polymer Functionalized, Nonporous Silica Nanoparticles for Quercetin Controlled Release
Source: Polymers (Basel). 2019 Dec 6;11(12):2026. doi: 10.3390/polym11122026 (PMC6960605; doi:10.3390/polym11122026)
Supplement: Supplementary file 1 [file polymers-11-02026-s001.pdf]

polymers

MDPI

Supporting information

PH-sensitive polymer functionalized nonporous silica nanoparticles for quercetin controlled release

Lin Xu [1], Hong-Liang Li [1,*] and Li-Ping Wang [2,*]

[1] College of Materials Science and Engineering, Qingdao University, Qingdao 266071, China; xulinbang@163.com
[2] College of Materials Science and Engineering, Liaocheng University, Liaocheng 252059, China
* Correspondence: lhl@qdu.edu.cn (H.-L.L.); wangliping5@163.com (L.-P.W.); Tel.: +86-532-8595-0767 (H.-L.L.); +86-635-8230-919 (L.-P.W.)

Materials and Methods

Materials

Silica nanoparticles (SNPs, pore size = 2.2 nm, pore volume = 0.8 cm^3/g, specific surface area = 155 m^2/g (measured by Brunauer−Emmett−Teller, BET) and 2.93 OH groups/nm^2 (calculated using eqn S1 reported by Bonilla-Cruz et al [1].) was dried for 8 h at 100 °C under vacuum before use. NaOtBu, phenothiazine, RuPhos Precat and RuPhos were purchased from Sigma-Aldrich. The 2-(diethylamino)ethyl methacrylate (PDEAEMA, AR) was obtained from Shanghai Chemical Reagent Plant and distilled under reduced pressure before use. α-Bromoisobutyryl bromide (BIBB), ethyl 2-bromo-2-methylpropionate (EBiB), triethylamine (TEA) and quercetin were acquired from Shanghai Aladdin Chemical Reagent Co. Ltd. 3-Aminopropyltriethoxysilane (KH-550), dimethylacetamide (DMA) and Tetrahydrofuran (THF) were purchased from Shanghai Sinopharm Chemical Reagent Co. Ltd. 10-Phenylphenothiazine (PTH) were synthesized according to the previously described literature [2]. Dulbecco's modified Eagle's medium (DMEM), 3-(4,5-dimethyl-thiazol-2-yl)-2,5-diphenyltetrazolium bromide (MTT) were purchased from Invitrogen Co. (Carlsbad, CA). L929 mouse fibroblast cells were obtained from the Shanghai Institute of Biochemistry and Cell Biology (SIBCB), Chinese Academy of Sciences. All other chemical reagents with analytical grade and used without further purification.

$$\left(\frac{\#OH}{nm^2}\right) = \frac{\left(Wt_{60}\text{-}wt_{800}\right)\times N_A}{M_{wH2O}\times S_{spec}\times wt_i}\times 10^{-18} \qquad \text{(S1)}$$

where wt_i is the initial sample weight before running TGA. S_{spec} is the specific surface area of SNPs.

Instrumental characterization

The molecular weights and polydispersities (PDI) of the polymers were determined via an alliance GPC 1515 (Waters 1515, Milford, MA, USA) gel permeation chromatographer (GPC) using THF as the eluent at a flow rate of 0.6 mL/min-1. Scanning electron microscopy (SEM) images were obtained using a scanning electron microscope (Merlin Compact, Zeiss, Germany) at 3.0 kV. ^1H NMR spectra were recorded on a 400 MHz (Varian Mercury Plus 400, Palo Alto, CA, USA) nuclear magnetic resonance instrument, using $CDCl_3$ as solvent and tetramethylsilane (TMS) as internal reference. The structures of samples were characterized by Nicolet-100 (Thermo Fisher Scientific,

Madison, WI, USA) Fourier transform infrared spectroscopy (FT-IR) from 400 cm^{-1} to 4000 cm^{-1}. X-ray photoelectron spectra (XPS) were performed on a Escalab 250xi spectrometer equipped with an Al Kα X-ray source (hv = 1486.6 eV). The thermal decomposition behavior of the materials was recorded on a STA 449C simultaneous DSC-TGA (Netzsch Instruments, Selb, Germany) thermo gravimetry analyzer (TGA) with a heating rate of 10 °C min^{-1} in nitrogen atmosphere. Differential scanning calorimetry DSC25 (TA Instruments, New Castle, USA) was used to measure the glass transition temperature (Tg) of PDMAEMA and SNPs-g-PDMAEMA from −70 to 150 °C at 5 °C/min. Transmission electron microscope (TEM) images were obtained using a JEOL JEM-1400 (JEOL. Ltd., Akishima, Japan) transmission electron microscope. The diameter of wall thickness and the average diameter of the hollow spheres were calculated using Intel Integrated Performance Primitives (ipp). Nitrogen adsorption-desorption isotherms curves were obtained using a Belsorp-Max (MicrotracBEL, Osaka, Japan), samples were degassed under vacuum at 120 °C before adsorption measurements. The release of quercetin in different PBS was monitored by UV-vis spectrophotometer UV-2200 (Beifen-Ruili, Beijing, china) at 367 nm, wavelength accuracy: ±0.25 nm. Microplate reader (Multiskan 1530) was purchased from Thermo Fisher Scientific Oy (Ratastie 2, FI-01620 Vantaa, Finland)

Synthsis of SNPs-Br

SNPs-Br was prepared according to the procedure described in previous study [3]. Specifically, 1 g of SNPs were dispersed in 10 mL of toluene, and then (2 mL, 8.561 mmol) of KH550 was added slowly with magnetic stirring at 90 °C for 11 h. The crude SNPs-KH550 was separated by centrifugation and washed completely by Soxhlet extraction with THF. The obtained SNPs-KH550 was dried at 40 °C under vacuum. XPS Calcd. (Atomic %): Si, 25.75; C, 21.40; N, 5.77; O, 47.09; Br, 0; N, 3.18 mmol g^{-1} (calculated according to the N atomic %) (Table 1).

SNPs-KH550 (1 g, 3.18 mmol NH_2 groups) were dispersed in THF (10 mL) containing TEA (2.5 mL, 17.986 mmol) and placed in a dried 50 mL flask with a magnetic stirring bar, and BIBB (4 mL, 32.360 mmol) was added dropwise into the reaction mixture at 0 °C for 11 h. After thoroughly rinsed with deionized water, ethanol and dried at 40 °C under vacuum, the product was obtained and denoted as SNPs-Br. XPS Calcd. (Atomic %): Si, 22.05; C, 25.16; N, 5.31; O, 44.22; Br, 3.27; Br, 1.67 mmol g^{-1} (calculated according to the Br atomic %) (Table 1).

Etching of silica with HF

The PDEAEMA on SNPs surface was recovered by chemical etching with a 20% HF aqueous solution for 24 h, and then the suspended particles of PMMA were centrifuged, washed with distilled water for three times and dried in vacuum oven. The molecular weight and distribution of the cleaved PDEAEMA from SNPs surface were list in Table S1.

Table S1 Molecular weight and distribution of the cleaved PDEAEMA from SNPs surface.

Sample	Mn [a]	Mw [a]	PDI [b]
PDEAEMA	16043	21206	1.32

Molecular weight of PDEAEMA determined by GPC using narrow PS standards, THF as the eluent.

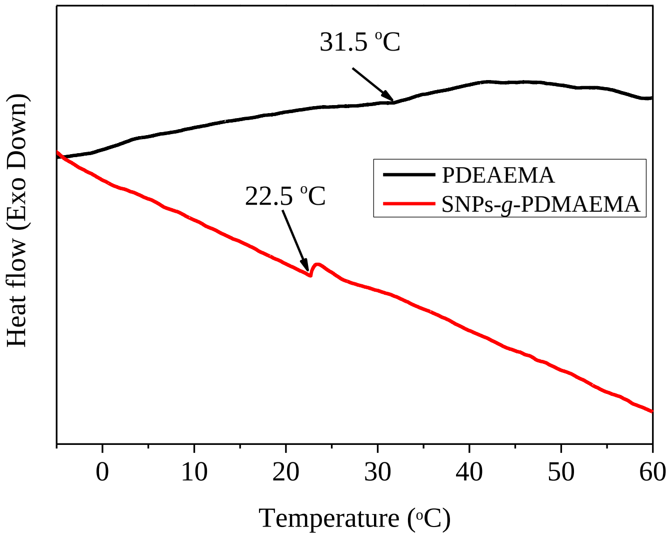

Figure S1 DSC curves of PDMAEMA and SNPs-*g*-PDMAEMA.

References

1. Arellano-Archán, E.; Alcalá, M. E.; Vega-Becerra, O.E.; Lara-Ceniceros, T. E.; Bonilla-Cruz, J. Optimizing the Coverage Density of Functional Groups over SiO_2 Nanoparticles: Toward High-Resistant and Low-Friction Hybrid Powder Coatings, *ACS Omega* **2018,** *3,* 16934–16944.
2. Treat, N. J.; Sprafke, H.; Kramer, J. W.; Clark, P. G.; Barton, B. E.; Alaniz, J. R.; Fors, B. P.; Hawker, C. J. Metal-free atom transfer radical polymerization, *J. Am. Chem. Soc.* **2014,** *136,* 16096-16101.
3. Yan, C.N.; Xu, L.; Liu, Q.D.; Zhang, W.; Jia, R.; Liu, C.Z.; Wang, S.S.; Wang, L.P.; Li, G. Surface-Induced ARGET ATRP for Silicon Nanoparticles with Fluorescent Polymer Brushes, *Polymers* **2019,** *11,* 1228.
